# Targeting NF-κB signaling cascades of glioblastoma by a natural benzophenone, garcinol, via *in vitro* and molecular docking approaches

**DOI:** 10.3389/fchem.2024.1352009

**Published:** 2024-02-16

**Authors:** Syed Mohd Danish Rizvi, Ibrahim A. Almazni, Mamdoh S. Moawadh, Zeyad M. Alharbi, Nawal Helmi, Leena S. Alqahtani, Talib Hussain, Ahmed Alafnan, Afrasim Moin, AbdElmoneim O. Elkhalifa, Amir Mahgoub Awadelkareem, Mohammad Khalid, Rohit Kumar Tiwari

**Affiliations:** ^1^ Department of Pharmaceutics, College of Pharmacy, University of Ha’il, Ha’il, Saudi Arabia; ^2^ Department of Clinical Laboratory Sciences, College of Applied Medical Sciences, Najran University, Najran, Saudi Arabia; ^3^ Department of Medical Laboratory Technology, Faculty of Applied Medical Sciences, University of Tabuk, Tabuk, Saudi Arabia; ^4^ Department of Biochemistry, College of Science, University of Jeddah, Jeddah, Saudi Arabia; ^5^ Department of Pharmacology and Toxicology, College of Pharmacy, University of Ha’il, Ha’il, Saudi Arabia; ^6^ Department of Clinical Nutrition, College of Applied Medical Sciences, University of Hail, Ha’il, Saudi Arabia; ^7^ Department of Pharmacognosy, College of Pharmacy, Prince Sattam Bin Abdulaziz University, Al-Kharj, Saudi Arabia; ^8^ Department of Clinical Research, Sharda School of Allied Health Sciences, Sharda University, Gautam Budh Nagar, India

**Keywords:** anti-cancer, benzophenone, garcinol, glioblastoma multiforme, NF-κB

## Abstract

Glioblastoma multiforme (GBM) is regarded as the most aggressive form of brain tumor delineated by high cellular heterogeneity; it is resistant to conventional therapeutic regimens. In this study, the anti-cancer potential of garcinol, a naturally derived benzophenone, was assessed against GBM. During the analysis, we observed a reduction in the viability of rat glioblastoma C6 cells at a concentration of 30 µM of the extract (*p < 0.001*). Exposure to garcinol also induced nuclear fragmentation and condensation, as evidenced by DAPI-stained photomicrographs of C6 cells. The dissipation of mitochondrial membrane potential in a dose-dependent fashion was linked to the activation of caspases. Furthermore, it was observed that garcinol mediated the inhibition of NF-κB (*p < 0.001*) and decreased the expression of genes associated with cell survival (Bcl-XL, Bcl-2, and survivin) and proliferation (cyclin D1). Moreover, garcinol showed interaction with NF-κB through some important amino acid residues, such as Pro^275^, Trp^258^, Glu^225^, and Gly^259^ during molecular docking analysis. Comparative analysis with positive control (temozolomide) was also performed. We found that garcinol induced apoptotic cell death *via* inhibiting NF-κB activity in C6 cells, thus implicating it as a plausible therapeutic agent for GBM.

## Introduction

Glioblastoma multiforme (GBM) represents the most commonly prevalent tumor of the human central nervous system, characterized by its aggressive malignancy and invasiveness. As per WHO classification, GBM fulfills all criteria for categorization amongst the deadliest grade of brain tumors (Grade IV) due to its malignancy and its mitotic and microvascular proliferation. It is observed that 60% of all brain tumors can be categorized into primary and secondary glioblastoma. Both types of GBM bear distinct genetic and epigenetic signposts and are histologically indistinguishable (Tiwari et al., 2021). Some characteristic pathophysiological features of GBM include hypoxia, necrosis, neo-angiogenesis, genetic deficiency, and poor prognosis, with an approximately 90% mortality rate (Phillips et al., 2018; Zhou et al., 2018). Ostrom et al. (2018) showed that 3.1 individuals are diagnosed with GBM per 100,000 individuals, with male populations more susceptible to this debilitating malignancy. Indeed, the heterogenicity with GBM tumor is a unique feature of this malignancy, coupled with its high invasiveness (Uddin et al., 2020). GMB is also reputed for its low survival time of only 15 months post-diagnosis. Intriguingly, this low survival rate is further correlated with high heterogenicity, unique properties of cell origin, and genetic mutations (Baid et al., 2020). The development of resistance to chemotherapeutics like temozolomide, along with post-surgical tumor relapse, is considered the main cause of the poor GBM prognosis. While current diagnostic advancements have resulted in a more precise classification of GBM tumor, a complete understanding of the disease’s development is still unclear.

Chronic inflammation is regarded as a major contributor in the onset of various diseases, including cancers such as GBM (Tiwari et al., 2021). The nuclear factor-κB (NF-κB) is a crucial regulator of inflammation during homeostatic and diseased conditions. It represents a family of transcription factors that are involved in regulating gene expression associated with several physiological responses, including cell proliferation, differentiation, inflammatory responses, apoptosis, and cell adhesion (Park and Hong, 2016). NF-κB-associated pathways are mostly activated by an array of stimuli including oncogenic stress, ultraviolet and ionizing radiation, DNA damage, growth factors, reactive oxygen species, and pathogen-related molecular patterns (Tiwari et al., 2021). Apart from its immunological role, chronic activation of NF-κB has now been established as a promoter of tumor initiation and progression by modulating key cellular processes such as angiogenesis, metabolism reprogramming, and tumor metastasis (Xia et al., 2014). Intriguingly, NF-κB-mediated inflammation may promote the proliferation of GBM tumor; this has further been reviewed by Shi et al. (2023). Avci et al. (2020) reported that administering NF-κB inhibitors alleviates the progression of tumor formation and the proliferation of patient-derived GBM cells. These findings indicate that NF-κB signaling can be considered a potential therapeutic target for clinically managing GBM.

In the quest for novel chemotherapeutic alternatives for cancer, natural bioactive compounds have gained considerable attention during the last two decades due to their reduced toxicity (Lin et al., 2020; Aziz et al., 2021; Huang et al., 2021; Kumar et al., 2021). Among these bioactive compounds, garcinol is a polyisoprenylated benzophenone abundantly present within the leaves and fruits of *Garcinia indica* and other members of *Garcinia* spp. found in the tropical regions of Africa and Asia (Jayaprakasha and Sakariah, 2002; Sung et al., 2019). Garcinol has been previously reported for several beneficial therapeutic attributes, including both *in vitro* and *in vivo* anti-inflammatory, anti-cancer, and anti-oxidant effects (Schobert and Biersack, 2019). Garcinol has also been reported to modulate apoptosis, arrest cell cycle progression, and prevent angiogenesis and metastasis in different pre-clinical models. This in turn indicates the probable efficacy of phytochemicals in restraining the progression of various cancers (Ye et al., 2014; Wang et al., 2020). Substantial molecular exploration of garcinol-mediated effects has also indicated that it mediates the inhibition of key cellular pathways associated with cancers, including ERK1/2, PI3K/AKT, STAT3, Wnt/β-catenin, and even the NF-κB pathway (Zhao et al., 2018). It was recently reported that the administration of garcinol with paclitaxel and cisplatin results in positive treatment outcomes in tumoral cells (Tu et al., 2017; Zhang et al., 2020). However, no report has yet investigated the effects of garcinol on pre-clinical and clinical models of GBM. Therefore, we aimed to explore the efficacy of garcinol on the proliferation of GBM cells and determine whether the NF-κB pathway was involved in the molecular mechanism of a garcinol-mediated anti-cancer effect on GBM progression. The experimental results demonstrated a crucial role of garcinol in the treatment of GBM, which may provide a new avenue for glioma research.

## 2 Materials and methods

### 2.2 Methods

#### 2.2.1 Cell culture maintenance

GBM C6 cells were purchased from the National Centre for Cell Science, Pune, India, and were cultured in DMEM-high-glucose medium. DMEM-high-glucose media, supplemented with 1% antibiotic–antimycotic cocktail and 10% FBS, was used in this study. All the imaging reported in the present study was conducted on a FLoid imaging station (Thermo Fischer Scientific, United States). The cells were passaged timely and were incubated at 37 °C in a humidified atmosphere with 5% CO_2_. All the fluorescent images were visualized and captured on a FLoid Imaging Station (Thermo Fischer Scientific, United States) at a fixed magnification of ×20 and scan area of 100 μm.

#### 2.2.2 Cell viability assay

The effect of garcinol in exerting cytotoxicity in C6 cells was assessed using MTT assay as described by Ahmad et al. (2022), although with subtle modifications. Approximately 1 × 10^4^ C6 cells were cultured in each well of the ELISA plate. This was followed by treatment with garcinol (10, 20, and 30 µM), and the cells were further incubated for another 24 h. Approximately 133.9 μM of temozolomide (Sigma-Aldrich, United States) was used as positive control (Poon et al., 2021). The stock of garcinol was prepared by dissolving it in DMSO at a concentration of 10 mM. The media of each well was then decanted, replaced with MTT stain (5 mg/mL), and the plate was further incubated for 3 h at 37 °C. Finally, the wells were supplemented with 100 µL DMSO, and the plate was gently agitated for 15 min. Absorbance of each well was recorded at 590 nm using a microplate reader (Bio-Rad, California, United States). Garcinol-mediated cytotoxic effects in C6 cells were expressed as cell viability percentages in comparison with the untreated C6 cells by using the following formula:

$$Cell\;viability\;\left(\%\right)=\frac{Absorbance\;of\;Garcinol\;treated\;C6\;cells}{Absorbance\;of\;untreated\;C6\;cells}\times 100.$$

Cells without any treatment served as the control and were subsequently used to compare the cytotoxicity of garcinol in C6 cells. The cytotoxic effect of garcinol was further evaluated on the cellular viability of normal murine lung alveolar macrophage (J774A.1) cells using the same protocol discussed above.

#### 2.2.3 Reactive oxygen species assay

Reactive oxygen species (ROS)-mediated oxidative stress levels within garcinol-treated C6 cells was assessed using DCFH-DA staining (Alshehri et al., 2022). We exposed 1 × 10^5^ C6 cells to the above-mentioned concentrations of garcinol for 12 h. Untreated cells were taken as a negative control, and C6 cells treated with 133.90 µM of temozolomide were used as a positive control. After incubation, the cells were stained with DCFH-DA (10 µM), and the plate was incubated for 30 min in the dark. Eventually, DCFH-DA-mediated green fluorescence was visualized, and the photomicrographs were captured from different garcinol-treated and/or untreated C6 cells. The changes in the level of DCFH-DA-mediated fluorescence in different garcinol treated cells were compared with the control cells.

Intracellular ROS levels were also quantified using DCFH-DA by nearly following the above-mentioned methodology. We seeded 1 × 10^5^ C6 cells in a 96-well black bottom which were exposed to garcinol (10, 20 and 30 µM) concomitantly, followed by 12 h of incubation under ambient tissue culture conditions. Subsequently, the cells were stained with 10 µM DCFH-DA, and the plate was left undisturbed for 30 min in the dark. Finally, DCFH-DA mediated fluorescence was measured at an excitation:emission wavelength of 485:528 nm using a Synergy H1 Hybrid Reader (BioTek, WA, United States). The observations were expressed as average DCFH-DA intensity percentages in comparison with the LPS stimulated positive control.

#### 2.2.4 DAPI assay

Alterations in the nuclear morphology of C6 cells were qualitatively assessed by DAPI staining as described by Alshehri et al. (2022). Approximately 1 × 10^4^ C6 cells were exposed to garcinol (10, 20, and 30 µM) in a 96-well plate and incubated for 24 h. After incubation, the cells were fixed with chilled methanol for 10 min. Untreated cells were taken as a negative control, and C6 cells treated with 133.90 µM of temozolomide were used as a positive control. Thereafter, the cells were cultured with DAPI stain (2 μg/mL) for 30 min at room temperature. Finally, the cells were visualized for DAPI-associated blue fluorescence.

#### 2.2.5 Caspase assay

Colorimetric assessment of caspase-9 and caspase-3 activities were evaluated in glioblastoma C6 cells as per Khafagy et al. (2022). During the assay, C6 cells were exposed to garcinol (10, 20, and 30 µM) in a 96-well plate. The cells were lysed and were centrifuged for 1 min (10,000×*g* at 4°C). We placed 50 μL of the supernatant on ice and supplemented it with an equal volume of reaction buffer constituted by 10 mM DDT. We added 4 mM of caspase-3 and caspase-9 substrates—DEVD-pNA, IETD-pNA and LEJD-pNA respectively—to each group and incubated them additionally for 10 min. Eventually, the absorbance of garcinol-treated and/or -untreated C6 cells was recorded at 405 nm. The results were expressed as caspase activity percentage (%) relative to untreated control C6 cells.

#### 2.2.6 Rhodamine assay

Rhodamine (Rh)-123 stain was used to study the effects of garcinol on the mitochondrial integrity of C6 cells as described in Shekh et al. (2022). We seeded 5 × 10^3^ C6 cells in each well of a 96-well plate, which were allowed to adhere overnight under standard culture conditions. Furthermore, the cells were exposed to various prescribed concentrations of garcinol for 24 h; post garcinol exposure, the cells were re-treated with 5 mg/mL of Rh-123 in the dark for 30 min. Untreated cells were taken as a negative control, and C6 cells treated with 133.90 µM of temozolomide were used as a positive control. Green fluorescent photomicrographs of C6 were recorded using an FLoid imaging station (Thermo Fischer Scientific, United States).

#### 2.2.7 Estimation of NF-κB via ELISA

A sandwich enzyme immunoassay kit was used for *in vitro* quantitative assessment of NF-κB in garcinol-treated and -untreated C6 cells. The amount of NF-κB were quantified in garcinol-treated and/or -untreated control C6 cells through ELISA kit (MBS2023542, MyBioSource, San Diego, CA, United States) following the manufacturer’s protocol.

#### 2.2.8 Real-time qPCR analysis

We seeded 1 × 10^6^ C6 cells and incubated them overnight for adherence. After the cells adhered, they were treated with the above-mentioned concentration for 4 h; post-treatment, the cells were exposed to varying stated concentrations of garcinol for 24 h. Subsequently, the total RNA was isolated from the control and the different treated C6 cells, and 2 µg of isolated RNA was subsequently used to synthesize cDNA using a Verso cDNA synthesis kit. The primers used in the study are shown in Table 1. Post-cDNA synthesis, qPCR reactions were performed using a DyNAmo ColorFlash SYBR Green qPCR Kit. Glyceraldehyde 3-phosphate dehydrogenase (GAPDH) was used as a housekeeping gene to normalize all quantifications. The comparative cycle threshold (CT) value was used to determine the variation in the expression of control and to evaluate the fold change in various treatment groups using the ^2−ΔΔCT^ method.

**TABLE 1:** Primers used during the qRT-PCR-based study.

S. no.	Target gene	Forward sequence	Reverse sequence	Reference
1	Bcl-XL	GCC​ACC​TAT​CTG​AAT​GAC​CAC​C	AGG​AAC​CAG​CGG​TTG​AAG​CGC	Ahmad et al. (2022)
2	Survivin	CCT​ACC​GAG​AAC​GAG​CCT​GAT​T	CCA​TCT​GCT​TCT​TGA​CAG​TGA​GG	
3	Bcl-2	GAT​TGT​GGC​CTT​CTT​TGA​G	CAA​ACT​GAG​CAG​AGT​CTT​C	Ahmad et al. (2022)
4	Cyclin D1	CCGTCCATGCGGAAGATC	GAA​GAC​CTC​CTC​CTC​GCA​CT	
5	GAPDH	GAA​ATC​CCA​TCA​CCA​TCT​TCC​AGG	GAG​CCC​CAG​CCT​TCT​CCA​TG	

#### 2.2.9 Western blot analysis

Post-garcinol treatment, C6 cells were pelleted and lysed in chilled RIPA buffer and were supplemented with a phosphatase and protease inhibitor (Sigma-Aldrich, Missouri, United States) cocktail for 30 min. A BCA protein estimation kit (Sigma-Aldrich, Missouri, United States) was used to quantify the protein concentration in various groups. The proteins were then separated through SDS-PAGE and subsequently transferred onto PVDF membranes (Millipore Corporation, Massachusetts, United States). Non-specific binding was prevented by incubating the PVDF membrane with blocking buffer, constituted by 5% non-fat milk for 2 h. Thereafter, the membrane was incubated overnight with primary antibodies at 4 °C. Post-incubation, the membrane was again incubated with horseradish peroxidase (HRP)-conjugated secondary antibodies for 2 h at room temperature and were then washed with TBST buffer. The expression of NF-κB, cleaved caspase-9, cleaved caspase-3, and surviving genes were evaluated by detecting changes in the chemiluminescence levels using an ECL kit (Sigma-Aldrich, Missouri, United States) following the manufacturer’s manual. Subsequently, the level of protein expression was normalized to relative internal standard and further quantified using ImageJ software (National Institutes of Health, Maryland, United States).

#### 2.2.10 *In silico* studies

Garcinol was docked using Autodock 1.5.7 (Rizvi et al., 2013). Protein structure (PDB ID: 1IKN) was modified by deleting water molecules and duplicate chains. The 3D structures of ligands, garcinol, and temozolomide (positive control) were obtained from the PubChem database. The centroid of the target protein was chosen as the binding pocket coordinate, and a grid box was placed within a cubic box of magnitude ×40 40 × 40 Å. Polar hydrogens and Kollman charges were added. Atoms were assigned AD4 type, and the molecule was saved in “pdbqt” format. For docking, search parameters like genetic algorithm and Lamarckian GA were used to save the output file.

#### 2.2.11 Statistical evaluation

The data are presented as mean ± SEM of three individual experiments, wherein each experiment was repeated thrice. Statistical significance between the control and different treatment groups was calculated using one-way ANOVA, followed by Dunnett’s *post hoc* test on GraphPad Prism software (Ver. 5.0). Significant differences between the treatment groups and the control were established if *p* < 0.05. The significance level was found to be *p < 0.05, **p < 0.01, and ***p < 0.001.

## 3 Results

### 3.1 Garcinol suppressed the proliferation of GBM C6 cells

**FIGURE 1:**
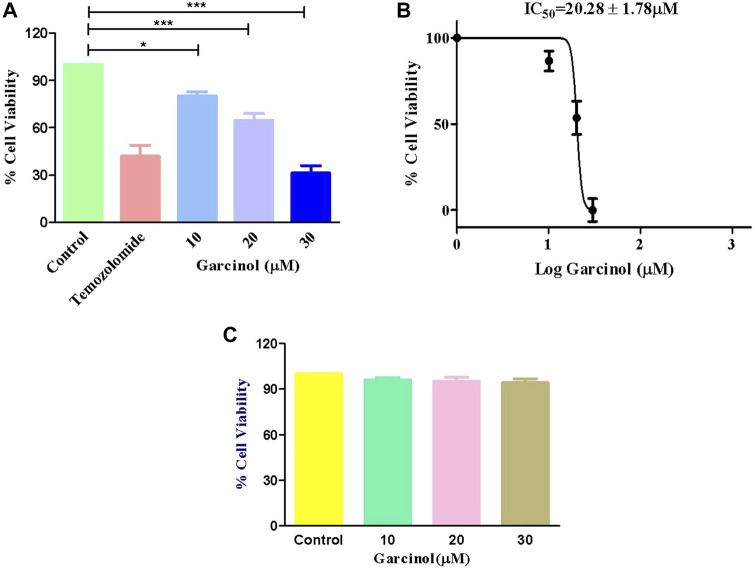
Garcinol-induced cytotoxic effects on murine glioblastoma C6 cells expressed as **(A)** percentage cell viability, **(B)** IC_50_ value of garcinol in C6 cells, and **(C)** garcinol effect on J774A.1 murine alveolar macrophages. **p < 0.01 and ***p < 0.001.

MTT assay was performed to determine whether garcinol could suppress the growth and proliferation of C6 cells. The cells were cultured with various doses of garcinol (5, 10, 20, and 30 µM) for 24 h. As demonstrated in Figure 1A, the cell viabilities of C6 cells decreased dose-dependently after treatment with increasing doses of garcinol. It was observed that garcinol reduced the cell viability of C6 cells to 90.80% ± 3.24%, 68.09% ± 5.42%, and 31.26% ± 3.71% at the indicated concentrations of 10, 20 and 30 µM, respectively. In the present study, temozolomide (positive control) was observed to reduce the viability of C6 cells from 100% (negative control) to 41.95% ± 2.40 after 24 h. The IC_50_ value of garcinol was found to be 20.28 ± 1.78 μM after 24 h of treatment (Figure 1B). Intriguingly, garcinol failed to induce any significant cytotoxic effects on J774A.1 cells at the above-mentioned concentrations (Figure 1C).

### 3.2 Garcinol augmented ROS-mediated oxidative stress

**FIGURE 2:**
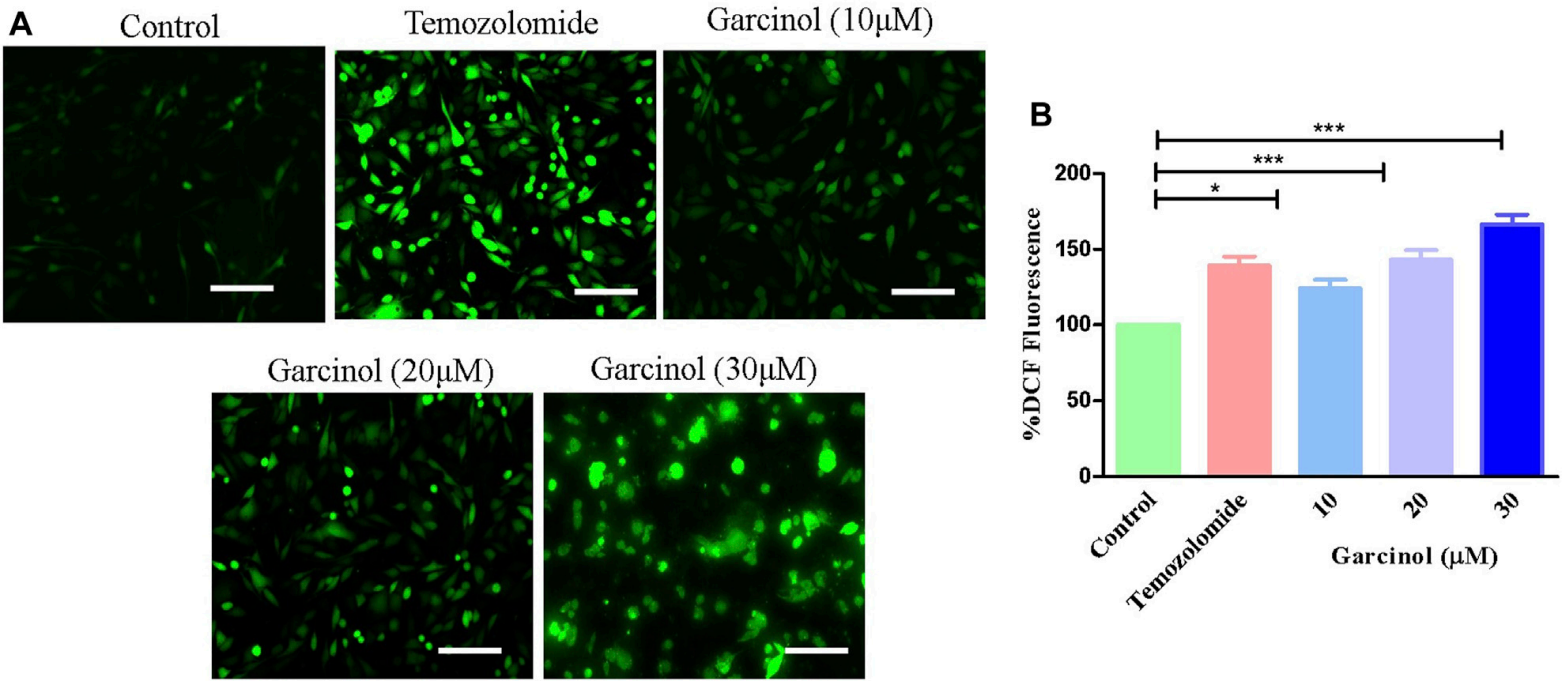
Efficacy of garcinol in instigating levels of **(A)** intracellular ROS and **(B)** quantification of ROS in garcinol-treated C6 cells. *p < 0.05, **p < 0.01, and ***p < 0.001.

Previous reports established that ROS plays a vital role in the occurrence and development of GBM, as well as in therapeutic strategies. Thus, targeting *via* ROS has been reviewed as an important interventional strategy against GBM (Liu et al., 2022). Fluorescence photomicrographs (Figure 2A) indicated enhanced levels of ROS-induced green fluorescence in garcinol-treated C6 cells in comparison to the positive control (temozolomide), indicating the significant generation of ROS. Subsequently, garcinol treatment increased intracellular ROS levels to 24.04% ± 4.82% (10 µM), 43.01% ± 5.16% (20 µM), and 66.42% ± 5.26% (30 µM) in comparison to temozolomide (44.04% ± 3.82%) in GBM cells (Figure 2B).

### 3.3 Garcinol mediated fragmentation and condensation in the nuclei of GBM C6 cells

**FIGURE 3:**
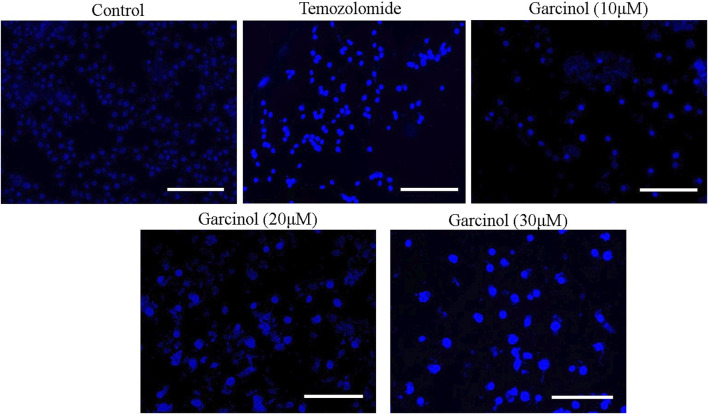
Garcinol-mediated effects on condensation of chromatin in C6 cells post-24 h exposure. Scale bar = 100 μm.

Garcinol-treated and/or -untreated C6 cells were dyed with DAPI to assess alterations within the nuclear morphology of C6 cells. The fluorescent micrographs (Figure 3) showed that C6 cells treated with garcinol exhibited marked chromatin condensation followed by nuclear shrinkage and subsequent formation of apoptotic bodies compared to the temozolomide (positive control), indicating characteristics of early apoptosis. These are well established attributes of activated apoptotic pathways. Thus, garcinol succeeded in altering the homeostatic nuclear morphology of C6 cells at significantly higher levels. Furthermore, the intensity of blue fluorescence in C6 cells was subsequently increased after treatment with 20 μM and 30 µM of garcinol.

### 3.4 Garcinol dissipated mitochondrial membrane potential in GBM C6 cells

**FIGURE 4:**
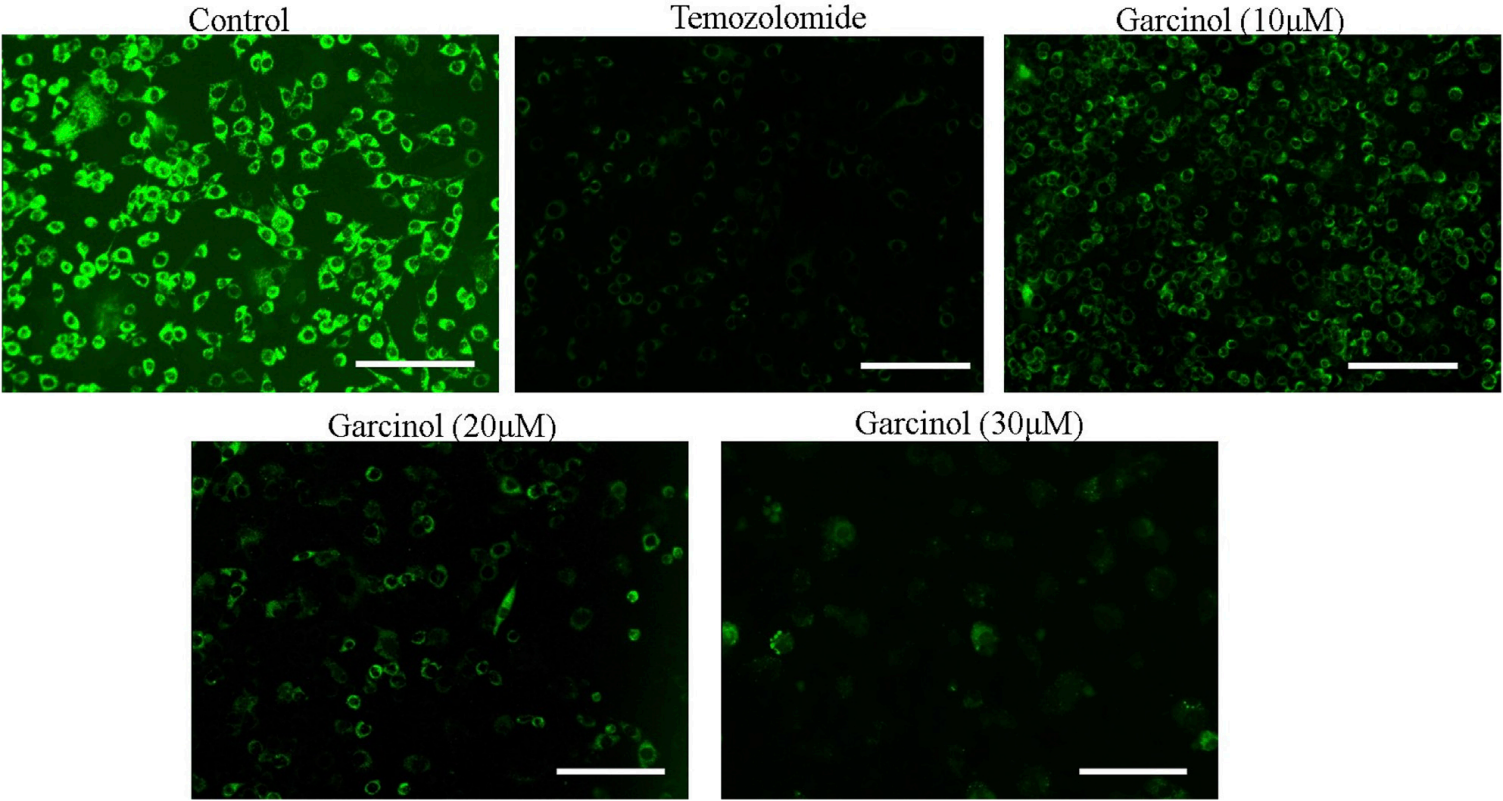
Garcinol-mediated effects dissipation of ΔΨm in C6 cells in a dose-dependent manner. Scale bar = 100 μm.

The dissipation of mitochondrial membrane potential (ΔΨm) is imperative to the activation of mitochondria-dependent apoptosis. To assess the effect of garcinol on ΔΨm of garcinol-treated C6 cells, mitochondrial potential specific Rh-123 dye was used. As demonstrated in Figure 4, the fluorescent photomicrographs evidently showed that exposure to varying concentrations of garcinol resulted in the reduced mitochondrial membrane potential of C6 cells, which in turn was again proportional to the increase in the concentration of garcinol that was comparable to the temozolomide (positive control).

### 3.5 Garcinol increased the activities of caspase-9 and caspase-3 in GBM C6 cells

**FIGURE 5:**
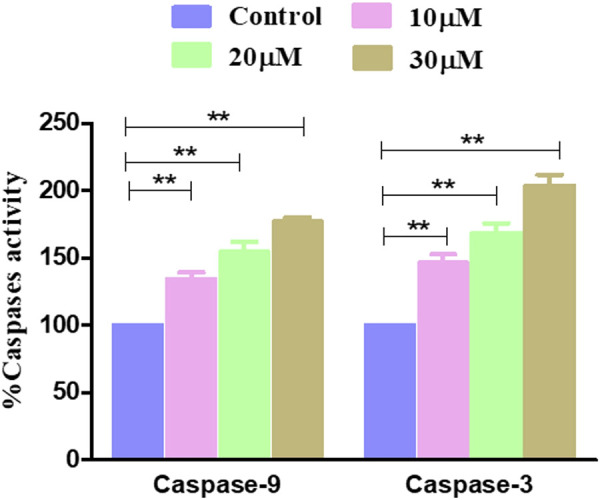
Activity levels of different caspases post-treatment with garcinol in C6 cells. *p < 0.05, **p < 0.01, and ***p < 0.001.

The effect of garcinol on the activities of caspase-9 and caspase-3 was investigated on rat glioblastoma C6 cells. The results showed that garcinol exposure dose-dependently triggered the activation of caspase-3. Concomitantly, caspase-9 activity levels were found to be significantly elevated by 34.31% ± 4.03% (10 μM), 54.73% ± 5.90% (20 μM), and 76.92% ± 2.62% (30 μM) compared to untreated control C6 cells upon exposure of the C6 cells to garcinol. Intriguingly, the activity of caspase-3 was found to be 46.36% ± 5.22%, 68.46% ± 5.95%, and 103.87% ± 6.68% compared to untreated control C6 cells at concentrations of 10, 20, and 30 µM (Figure 5), respectively.

**FIGURE 6:**
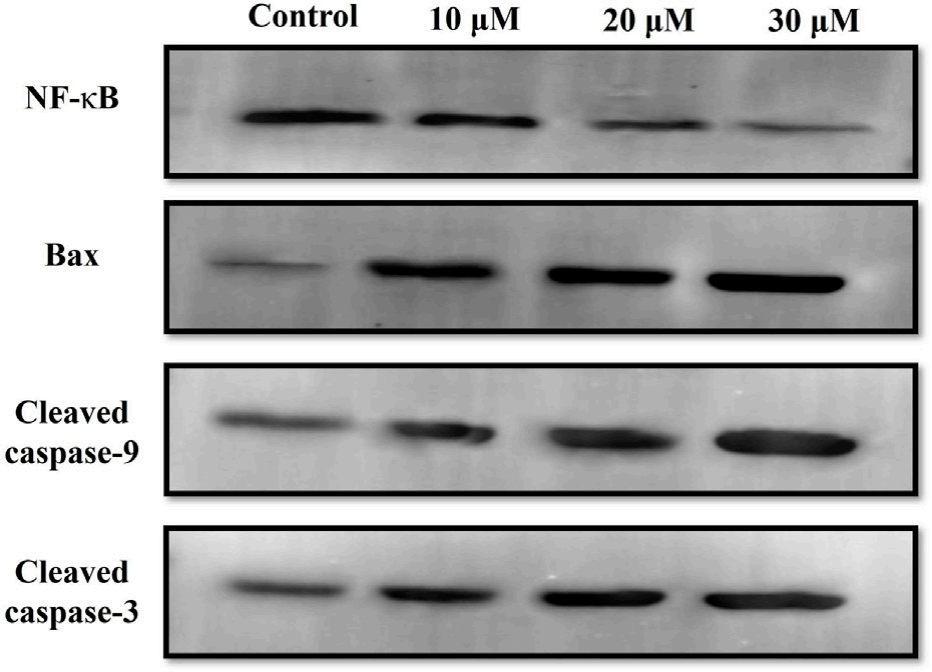
Expression levels of NF-κB along with various key genes involved in regulating the apoptotic cell death within C6 cells exposed to garcinol.

**FIGURE 7:**
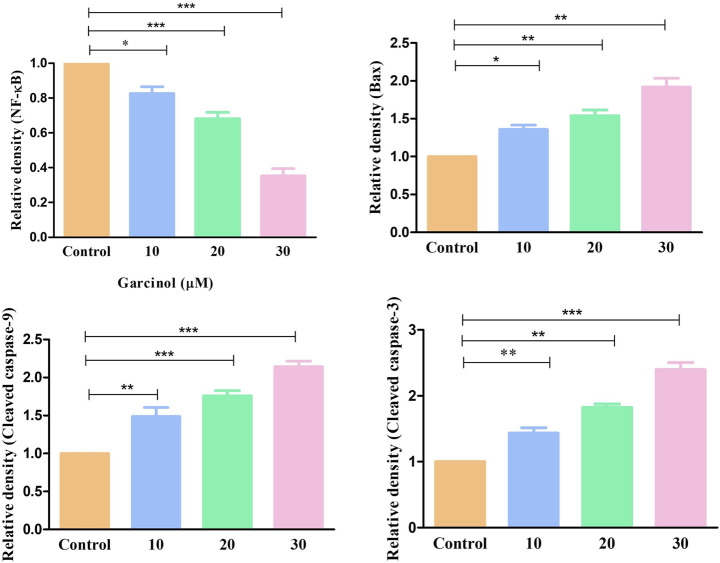
Quantification of NF-κB along with various key genes involved in regulating the apoptotic cell death expressed within C6 cells exposed to garcinol. *p < 0.05, **p < 0.01, and ***p < 0.001.

Western blot was also performed to ascertain the role of caspase activation in garcinol-induced apoptosis in C6 GBM cells (Figures 6; 7). The effect of garcinol was thus studied on the expression levels of cleaved caspase-3 and caspase-9. As demonstrated in Figures 6 and 7, garcinol could substantially elevate the expression levels of cleaved caspase-3 and caspase-9. In addition, garcinol also increased the expression level of Bax protein in C6 cells. Thus, garcinol could function as an important and specific mediator facilitating the activation of multiple caspase cascades.

### 3.6 Garcinol mediated inhibition of NF-κB and its associated genes

**FIGURE 8:**
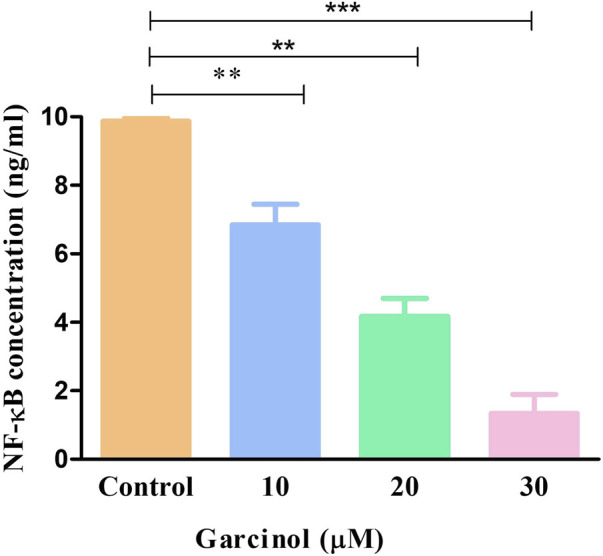
Levels of NF-κB in murine glioblastoma C6 cells treated with varying garcinol concentrations for 24 h. *p < 0.05, **p < 0.01, and ***p < 0.001.

NF-κB is a transcription factor that regulates multiple genes which mediate different cellular activities such as proliferation, differentiation and the survival of cancer cells. In GBM, different stimuli are responsible for the abnormal activation of NF-κB. Consequently, in order to investigate the underlying anti-cancer and apoptotic mechanism of garcinol in C6 cells, NF-κB levels were estimated using ELISA-based assay. The results demonstrated that garcinol decreased the levels of NF-κB to 1.33 ± 0.45 ng/mL in comparison with the untreated cells (Figure 8).

**FIGURE 9:**
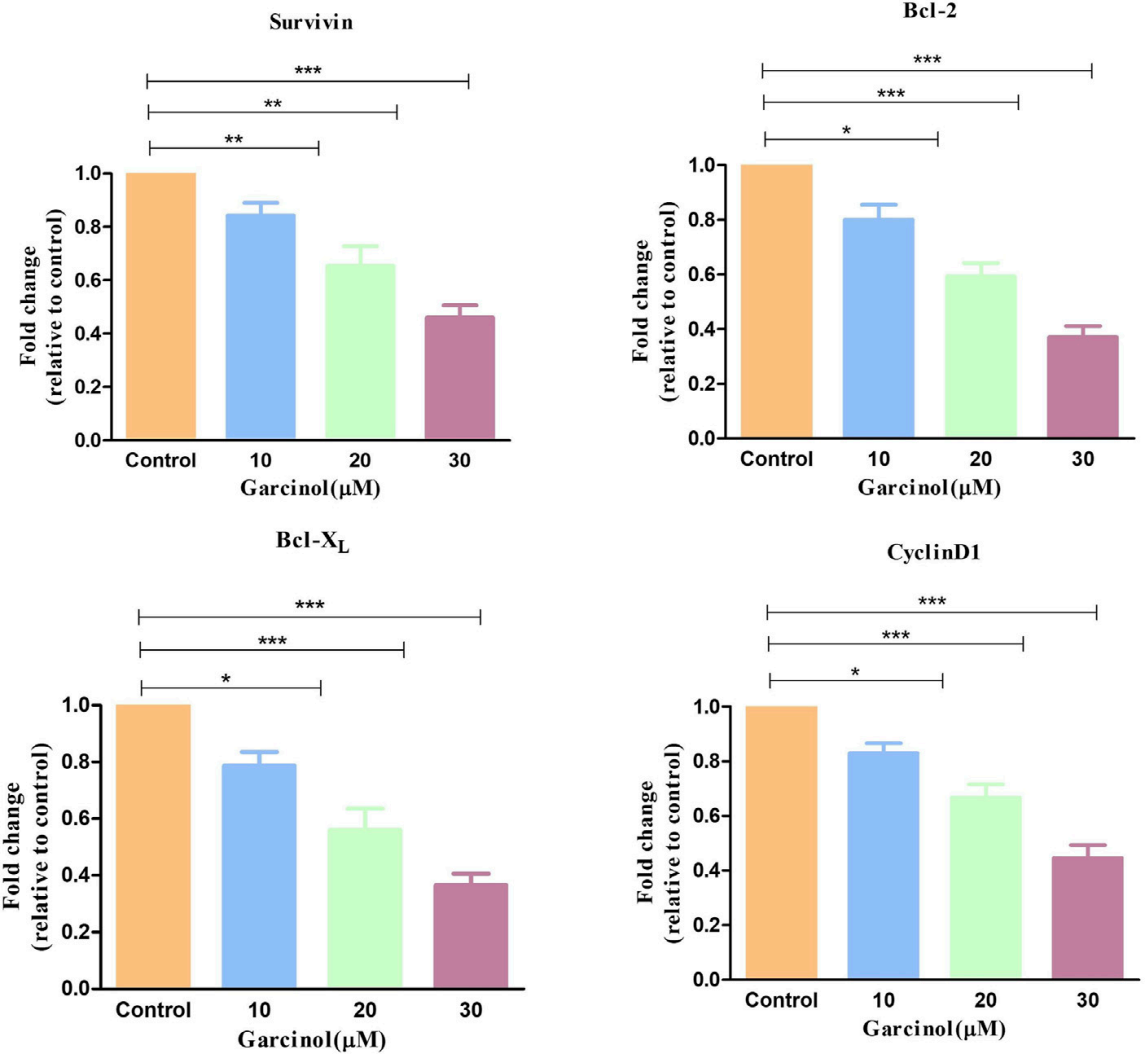
qRT-PCR observation indicating the expression of key apoptosis-controlling genes in murine glioblastoma C6 cells treated with varying concentration of garcinol. *p < 0.05, **p < 0.01, and ***p < 0.001.

NF-κB is basically regarded as a pro-survival factor that induces the expression of genes that promote cell survival and proliferation. Proteins regulated by NF-κB in GBM which act in this manner include Bcl-2, Bcl-XL, survivin, and the inhibitor of apoptosis proteins. qRT-PCR-based studies have shown that treatment with increasing doses of garcinol downregulated the level of survivin, Bcl-2, and Bcl-XL mRNA by 0.84 ± 0.03-, 0.65 ± 0.06-, and 0.46 ± 0.03-fold, 0.83 ± 0.05-, 0.54 ± 0.05-, and 0.43 ± 0.07-fold, and 0.82 ± 0.04-, 0.56 ± 0.06-, and 0.36 ± 0.03-fold, respectively, compared to the control cells (Figure 9). Subsequent qRT-PCR-based assessment revealed the modulatory role of garcinol on the specific mRNA expression of downstream target genes of NF-kB in C6 cells. As presented in Figure 9, garcinol reduced the mRNA levels of cyclin D1 by 0.83 ± 0.02, 0.71 ± 0.06, and 0.44 ± 0.03-fold at 10, 20, and 30 µM concentrations, respectively. This indicates that garcinol effectively alters the expression of genes associated with the cellular proliferation of C6 cells.

### 3.7 Molecular docking studies

**FIGURE 10:**
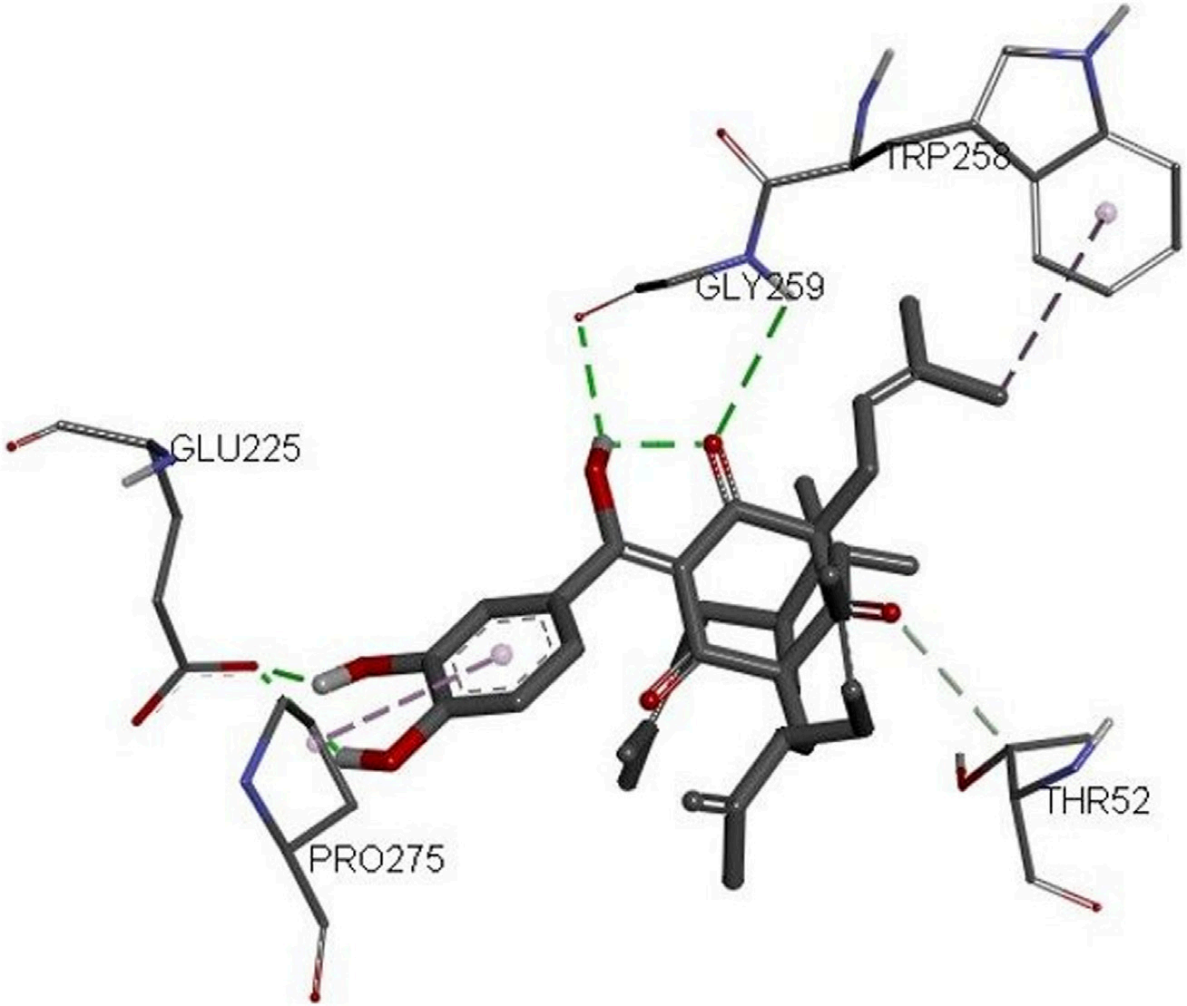
3D structure of the docked complex of NF-κB and garcinol.

**FIGURE 11:**
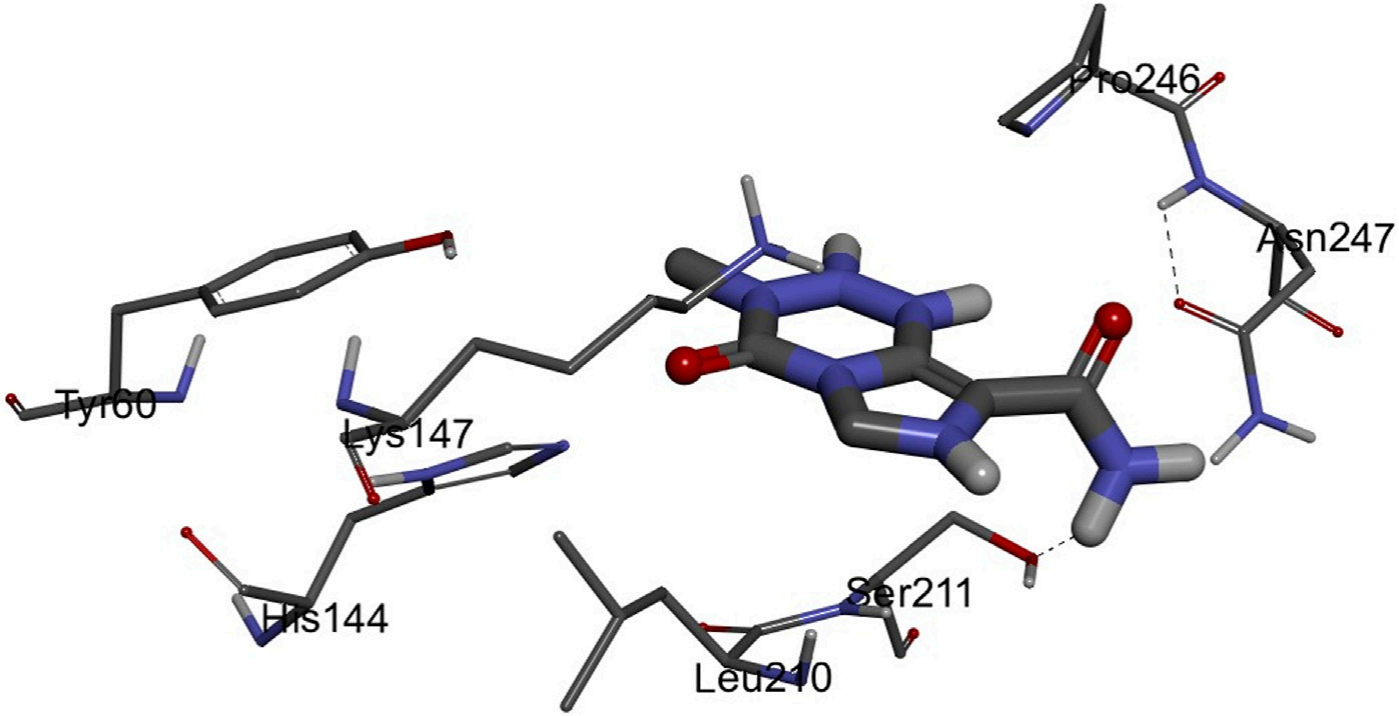
3D structure of the docked complex of NF-κB and temozolomide.

The 3D structures of NF-κB, temozolomide, and garcinol are shown in Fig. 1S. The results of molecular docking studies illustrated that the binding energy of garcinol to NF-κB was found to be −4.36 kcal/mol, whereas the binding energy of temozolomide with NF-κB was −4.72 kcal/mol. The residues A: Pro^275^ and F: Trp^258^ were involved in pi-alkyl bonding, A: Thr^52^ d in CH bonding, and F: Gly^259^ and A: Glu^225^ in hydrogen bonding during the interaction between garcinol and NF-κB (Fig. 2S and Figure 10). On the other hand, Ser^211^ residue was involved in hydrogen bonding, and Tyr60, His144, Lys147, Leu210, and Asn247 in hydrophobic interaction during the binding of temozolomide with NF-κB (Figure 11).

## 4 Discussion

Despite their effectiveness, current chemotherapeutics are usually restricted in their usage owing to limitations that include high manufacturing costs and significant side effects. NF-κB inhibitors currently in use also face similar challenges, thus necessitating the exploration of natural NF-κB inhibitors (Baud and Karin, 2009). Activated NF-κB ensures a profound pro-inflammatory environment within GBM, favoring the unchecked proliferation of tumor cells. Intriguingly, this pro-inflammatory milieu within GBM can be considered a plausible therapeutic target. Several studies have been conducted to elucidate the efficacy of various anti-inflammatory compounds for the clinical management of GBM, but no consolidated conclusions have yet been reached (Puliyappadamba et al., 2014).

Among the various bioactive compounds present within *G. indica*, garcinol is considered a major bioactive constituent. Garcinol has widely been reported to possess intrinsic therapeutic benefits, such as anti-cancer and anti-oxidant properties, and for its structural similarities with curcumin (Saadat and Gupta, 2012). The anti-cancer effect of garcinol was studied against colon cancer by Hong et al. (2007). They reported that garcinol substantially decreases the growth of cancer cells with reduced toxicity against normal healthy cells. Intriguingly, garcinol was also found to impede NF-κB/STAT-3 signaling cascade *in vitro* and also substantially reduce breast cancer growth in NOD-SCID mice (Ahmad et al., 2012). Such evidence therefore presents a strong rationale for investigating the plausible anti-GBM effect of garcinol. In addition, garcinol inhibits the proliferation of gastric cells by suppressing cell invasion and migration and it promotes cell apoptosis. Subsequent studies have demonstrated that garcinol is likely to exhibit these effects by suppressing the PI3K/AKT signaling pathway and decreasing the expression of cyclin D1, MMP2, and MMP9 in HGC-27 cells. Garcinol may thus play a crucial role in gastric cancer and may serve as a novel therapeutic agent (Zheng et al., 2020). Another study found that garcinol holds anti-cancer potential in combination with cisplatin in ovarian cancer cells (Zhang et al., 2020).

The present study observed that garcinol suppressed the growth and proliferation of C6 cells. During the MTT assay, garcinol also significantly reduced the viability of C6 cells. We thus concluded that garcinol inhibited the growth of C6 cells, suggesting the probable cytotoxic and anti-cancer effects of garcinol against C6 cells. Furthermore, it was also observed that garcinol was potentially tolerable or non-cytotoxic against normal cell line J774A.1 cells. The findings were subsequently confirmed by photomicrographs, illustrating characteristic alterations in the morphological features of garcinol-treated C6 cells closely associated with cell death.

Augmentation of ROS was found to correlate with the activation of DNA damage beyond repair, resulting in apoptotic cell death (Yang et al., 2017). Treatment with garcinol also induced significant nuclear fragmentation and chromatin condensation, observed through DAPI staining. ROS-mediated damaging effects on DNA have been substantially associated with the onset of apoptosis (Su et al., 2013; Zorov et al., 2014).

The present study observed that garcinol significantly escalated the ROS generation, which culminated in a series of events, resulting in cell death in C6 cells. Mitochondria are known to be the main repertoire for the production of ROS (Zorov et al., 2014), and excess ROS production by mitochondria has resulted in opening of the mitochondrial permeability transition pores facilitated by the dissipation of ΔΨm. The opening of these pores thus resulted in augmented levels of cytosolic cytochrome-c (Zhang et al., 2015). Therefore, the present study demonstrates that treatment with garcinol increases the ROS levels and substantially dissipates the mitochondrial membrane potential in C6 cells. Furthermore, loss of mitochondrial membrane potential is also linked to the activation of caspases, which are known as critical regulators of apoptosis (Zhu et al., 2012). The results suggest that garcinol mediates the substantial activation of caspase-9 and -3 within C6 cells, thus instigating apoptotic cell death via the activation of the intrinsic apoptotic pathway.

Overexpression of NF-κB pathway has been reported in GBM, which is also known for exhibiting antiapoptotic activity (Raychaudhuri et al., 2007). NF-κB is typically regarded as a pro-survival factor, accountable for inducing the expression of genes that expedite cell survival and proliferation. Proteins such as Bcl-2, Bcl-XL, and survivin are regulated by NF-κB in GBM (Koul et al., 2006). The present study demonstrated that garcinol substantially decreased the gene expression of these antiapoptotic proteins in C6 cells. Cyclin D1 is another target protein of NF-κB that is involved in survival and proliferation (Witzell et al., 2010) and is responsible for poor prognosis in GBM patients (Sallinen et al., 1999). The findings from qRT-PCR-based investigations indicate the competency of garcinol in decreasing the gene expression of cyclin D1 in glioblastoma cells. Thus, treatment with garcinol can significantly inhibit NF-κB signaling and, critically, modulate its associated target genes that are involved in cellular proliferation and resistance to apoptosis in C6 cells.

Furthermore, molecular docking analysis of garcinol with NF-κB confirmed the involvement of important amino acid residues such as Pro^275^, Trp^258^, Glu^225^, and Gly^259^ (Huxford et al., 2002; Cai et al., 2021; Ravish et al., 2023). Cai et al. (2021) revealed the role of Glu^225^ and Gly^259^ amino acids during quercetin: NF-κB interactions. Recently, Ravish et al. (2023) indicated a role for Pro^275^ and Glu^225^ during pyrimidine derivative interactions with NF-κB, whereas Huxford et al. (2002) documented the crucial role of Trp^258^ in the binding and specificity of NF-κB to Iκ-Bα. On the other hand, the binding energy of temozolomide (positive control) interaction with NF-κB was better than garcinol. However, none of these crucial amino acids were involved in temozolomide interaction with NF-κB. Thus, the molecular docking results further supported the *in vitro* outcomes.

*In silico* findings exhibited the strong binding affinity of garcinol with NF-κB protein. Furthermore, these results supported real time PCR results and provided a strong rationale for why garcinol significantly inhibits NF-κB signaling in GBM cancer cells.

While pre-clinical explorations have produced promising outcomes, the development of garcinol as an anti-cancer agent in therapeutics is mainly limited by poor understanding of the systematic pharmacokinetic evaluation of garcinol. It is necessary to determine the effects of the appropriate dosage on its pharmacodynamic attributes. In addition, earlier studies lacked a regulatory network for the downstream target genes of NF-κB. Subsequent studies should focus on enhancing the integrity of the experiment and understanding the molecular basis of the anti-GBM potential.

## 5 Conclusion

The present study demonstrated that garcinol exerts anti-cancer efficacy by suppressing the growth and mediating programmed cell death of human GBM C6 cells by impeding the activation of NF-κB. The results were corroborated by *in silico* investigation. These results further necessitate the need for preclinical and clinical research regarding the potential use of garcinol as an anti-GBM drug. Subsequent in-depth analysis of the exact mechanism of apoptosis induced by garcinol in GBM cells is also required. Additionally, subsequent studies are needed to investigate the anti-cancer effect of garcinol with existing chemotherapeutic drugs. Furthermore, the efficacy of garcinol was studied on a limited range of cancer cell lines, so their spectrum of activity should be expanded. In some instances, the potency of garcinol is limited by its poor bioavailability. Thus, researchers should lay substantial emphasis not only on the efficacy of compounds but also on drug delivery systems in order to overcome pharmacokinetic issues while studying derivatives with a high degree of biological efficacy and availability.

## Data Availability

The original contributions presented in the study are included in the article; further inquiries can be directed to the corresponding authors.
