# Supplementary figures and images for: Targeting NF-κB signaling cascades of glioblastoma by a natural benzophenone, garcinol, via in vitro and molecular docking approaches
Source: Front Chem. 2024 Feb 16;12:1352009. doi: 10.3389/fchem.2024.1352009 (PMC10904546; doi:10.3389/fchem.2024.1352009)

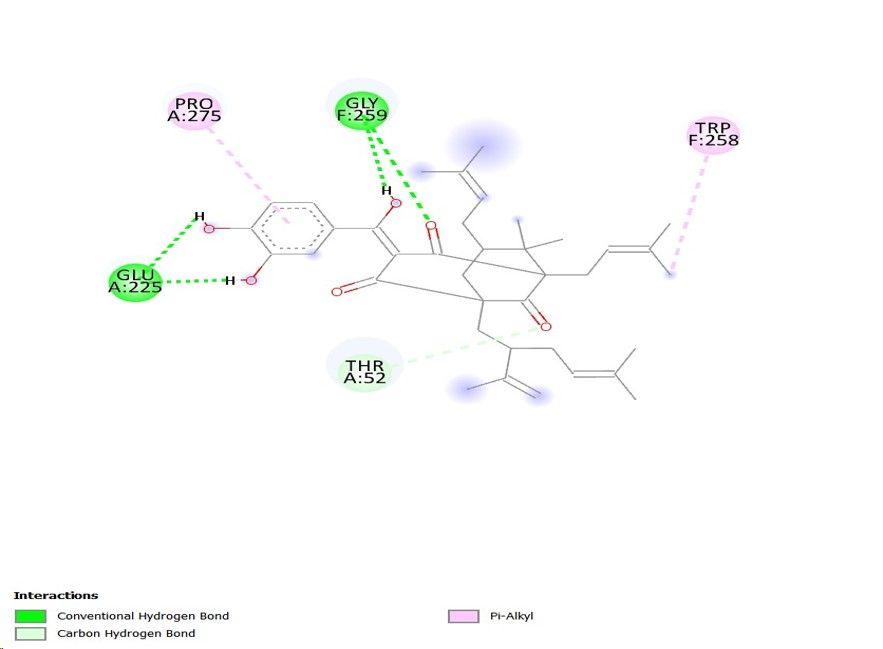

Supplement: Supplementary file 1 [file Image2.TIF]

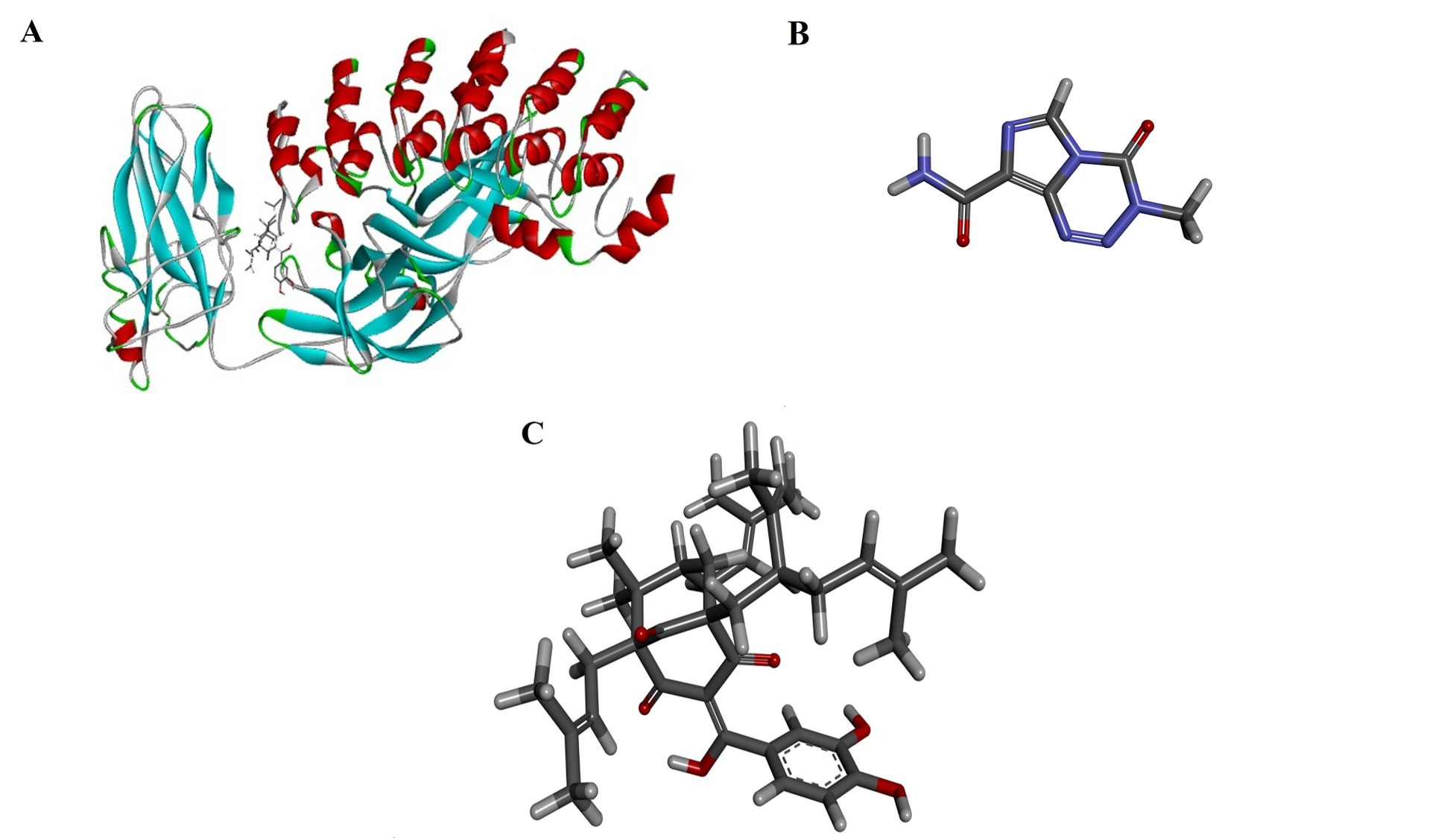

Supplement: Supplementary file 2 [file Image1.TIF]
